# Essays on Hypochondriacal and Other Nervous Affections

**Published:** 1830-07-01

**Authors:** 


### RETROSPECTIVE REVIEW.

HAVING cleared our score with the current publications of the day, we shall now try back on our retrospective plan, and present our readers with the analyses of some important works which are unknown to the juniors of the profession and forgotten by too many of the seniors. It is surprising, indeed, how soon even the best productions are buried or swallowed up in the vast torrent of works which issue from the press—so that it is only by recalls like these, that they are preserved from annihilation or oblivion! We are convinced, therefore, that an occasional reminiscence of this kind will be a useful labour—more useful perhaps than a search after what is called novelty.

ESSAYS ON HYPOCHONDRIACAL AND OTHER NERVOUS AFFECTIONS. By *John Reid*, M.D. Member of the Royal College of Physicians, London; and late Physician to the Finsbury Dispensary. 8vo. pp. 272.

So close is the connexion of the mind and the body, and such is their mutual influence in action or suffering, that in no possible case of human life, can the one be affected without producing some impression on the feelings of the other. Yet this truth, which has never been denied even by the sternest stoic, or the most subtle metaphysician, has attained universal assent, rather as an article of sensation than of science; as being the compulsion of experience more than the result of enquiry. Some men may call in question the liberty of the human will; and others may weave a fine spun chain of arguments against the existence of matter and the reality of an external world: but after all, whether our thoughts are strung together by necessity, and our movements are impelled by causes over which we have no control; whether the forms that occasion pleasure or pain are substantial or ideal, still the conclusion is the same, as it relates to the sympathy by which the mental and corporeal faculties mutually operate to the ease or disquiet of the entire system. It is, therefore, mortifying to the pride of man's wisdom, that in an age which, above all others, has pushed with the greatest effect the energy of philosophical investigation into the hidden mysteries of Nature, which has traced the minutest essences through a variety of modifications to their elemental principles, with an assiduity that claims applause, and a success that commands admiration; still, after all, and to abate the swell of vanity arising from this consciousness of intellectual superiority, man is made to confess that he knows more of the world around him, than of that which he carries within his own compact form. They who, more than others, are obliged to study the constitution of the human frame, and to extend their inquiries into a vast variety of scientific objects, that they may be qualified to render that study complete and beneficial, even the professors of the noblest art that can engage the time and the talents of man, are in like manner compelled to acknowledge their total inefficiency to account for the aberrations that so frequently disturb the machine with which they are most acquainted. This *crux medicorum* is the more distressing, because while it urges application, it confounds the judgment; and at the same time that it calls for the discovery of a remedy, it baffles hope, in being under the necessity of leaving that to chance which can never be determined by any certain principle of operation, or be regulated by any rule of practice. Without presuming to advance that the awful malady of Insanity, in all its shades of gradation, can never become so precisely defined as to admit of medical management with a probability of success; we must at least be allowed to say, that hitherto our acquaintance with mental diseases has gone little further than to an observation of general causes and effects, over which professional skill may exert its best efforts in vain. With this impression on our minds, we took up the present volume, in the expectation of seeing much ingenuity wasted in an attempt to remove that opprobrium of medical science, which the subject of insanity has so long proved. We were prepared, indeed, from what we had with pleasure read in the periodical reports of the author, to meet with some acute and lively remarks on extraordinary cases, as also with sagacious counsel in regard to the means employed for the relief of persons affected by nervous disorders. But we had no thought that in a book bearing an unassuming title, and upon an unpromising topic, would be found so many new lights, in the simple form of hints, on the various excitements to mental disease, and upon the injudicious manner in which they are too commonly treated. That the author has been prevented from fulfilling his original intention of publishing a systematical treatise on the subject, is rather a matter of congratulation than otherwise; as, by throwing out his observations in the form of Essays, he has rendered his work more likely to become popular and beneficial, by bringing it immediately to the view of those who would be alarmed at the thoughts of perusing any thing like a theoretical or argumentative performance.

The Essays are twenty-seven in number; and although they have not the formality of a connected arrangement, yet, as if the maxim of Horace had been under contemplation, the reader will find in his progress, that the order could not have been better disposed, even to constitute a train of leading principles.

The first Essay is "On the Influence of the Mind on the Body," in which consciousness, as the peculiar faculty of man, is set forth in strong and elegant language. The following remark, at the close, strikes us as equally new and important.

"The class of persons whose lives are devoted to mere manual labour, especially the more indigent part of them, are, to a certain extent, distinguished by the character of their diseases, as well as that of their other evils. They differ from the higher orders, less perhaps in the actual quantity, than in the glaring and obtrusive colour of their calamities.

"There is no person, perhaps, who is apt to form so low an estimate of the value of human existence, as a medical man practising amongst the poor, especially amongst the poor of a great city. But it is not impossible that he may exaggerate the excess of their sufferings, by combining, as it is natural for him to do, their external state with those feelings which he has acquired from very different circumstances and education. As the horrors of the grave affect only the living, so the miseries of poverty exist principally, perhaps, in the imagination of the affluent. The labour of the poor man relieves him at least from the burden of fashionable ennui, and the constant pressure of physical inconveniences, from the more elegant, but surely not less intolerable distresses of a refined and romantic sensibility. Even those superior intellectual advantages of education, to which the more opulent are almost exclusively admitted, may, in some cases, open only new avenues to sorrow. The mind in proportion as it is expanded, exposes a larger surface to impression."

Essay II. On "The Power of Volition," exhibits some curious cases, wherein several persons have been known to "possess power not only over the feelings and faculties of the mind, but likewise over what are called the involuntary muscles, and even the blood-vessels of the body." But, amusing as this part of the Essay is, what the author has observed on the inhumanity of treating hypochondriacs with ridicule, is better adapted to improve the feelings and to regulate the practice of men.

"No one was ever laughed or scolded out of hypochondriasis. It is scarcely likely that we should elevate a person's spirits by insulting his understanding. The malady of the nerves is in general of too obstinate a nature to yield to a sarcasm or a sneer. It would scarcely be more preposterous to think of dissipating a dropsy of the chest, than a distemper of the mind by the force of ridicule or rebuke. The hypochondriac may feel indeed the edge of satire as keenly as he would that of a sword; but although its point should penetrate his bosom, it would not be likely to let out from it, any portion of that noxious matter by which it is so painfully oppressed. The external expression of his disorder may be checked by the coercive influence of shame or fear; but in doing this, a similar kind of risque is incurred as arises from the repelling of a cutaneous eruption, which, although it conceal the outward appearance, seldom fails still more firmly to establish the internal strength, to increase the danger, and to protract the continuance of the disease. By indirect and imperceptible means the attention may, in many instances, be gently and insensibly enticed, but seldom can we with safety attempt to *force* it from any habitual topic of painful contemplation. In endeavouring to tear the mind from a subject to which it has long and closely attached itself, we are almost sure to occasion an irreparable laceration of its structure."

In the third Essay, "On the Fear of Death," the reader will meet with much excellent reasoning, to dispel apprehensions which are, in themselves, more tormenting than the object of dread. The author deprecates all tendency to encourage despondency; and among the rest, he shows the fatal effects of predictions of death.

"In dangerous maladies, the person in whom there is the least fear of dying, has, other circumstances being the same, the fairest chance to survive. Men, in critical situations, are apt to be overwhelmed by their terrors; they are drowned by their too eager struggles to emerge; they would keep afloat, if they remained quiescent."

The effects of Pride on the mind in producing mental derangement, constitute the subject of the fourth Essay; in which we were much pleased with this judicious discrimination in the mode of treating different persons.

"The humbly nervous ought to be treated with the most encouraging respect, and with the most courtier-like attention. We should endeavour, by expressions of an extraordinary regard for them, to supply the want of satisfaction which they are apt to feel with themselves. On the other hand, a haughty imbecility ought to be met by a management that is calculated to depress the patient in his own eyes, and to sober a spirit that may have been intoxicated by draughts of a servile or treacherous adulation."

The next Essay is on "Remorse," which, beyond doubt, is one of the most dreadful of all diseases when it has become fixed in the mind, and the most difficult to cure. But it should be considered, and the author has prudently laid great stress upon the fact, that remorse is not always occasioned by actual misconduct. It is in truth, perhaps, no less frequently the suffering of a tender and upright mind, than of a guilty conscience. Of this we have in addition to some well known cases, the following; which came under the observation of the author himself.

"It is not very long since I had a professional opportunity of knowing something of the morbid history of a man, who had succeeded to a peerage, and an immense estate, by the death of an elder brother, with whom he had not been upon good terms for some years previous to that event. The unfortunate heir to the title and domains so severely reproached himself for that suspension of fraternal amity, with regard to which he was altogether innocent, that he sunk into a profound melancholy, from which I have reason to believe nothing has hitherto been able to rouse him.

"I knew another person, who, although his life had been signalized by the most active and successful exertions in behalf of his fellow creatures, was affected with a despondency, the burden of which was, that he had been all along a useless member of society, and that the talents which had been given him had produced nothing in his hands. Under the influence of this imagination, he expressed a kind of horror as well as shame, at the prospect of giving up a stewardship, the duties of which he had, as he thought, so unfaithfully discharged."

"Not many months ago, I had an opportunity of knowing an instance of the melancholy effect of remorse, where the feeling, although not altogether without foundation, was unduly aggravated by an accidental association of occurrences.

"A young lady was one morning requested by her mother to stay at home; notwithstanding which, she was tempted to go out. Upon her return to her domestic roof, she found that the parent, whom she had so recently disobliged, had expired in her absence. The awful spectacle of her mother's corpse, connected with the filial disobedience which had almost immediately preceded, shook her reason from its seat, and she has ever since continued in a state of mental derangement."

It is well observed, at the commencement of the sixth Essay, that "an hypochondriac should be a hermit in abstinence, but not in solitude." This subject of seclusion from the pleasures of society is ably treated, and the danger of retirement to those who have been accustomed to business is clearly shewn and supported by proofs. Yet a due caution is introduced with regard to the choice of society; for, as it is properly observed,

"We are not perhaps sufficiently aware that nervous complaints are, through the medium of sympathy, scarcely less infectious than febrile diseases. Amongst many other instances illustrative of this opinion, I particularly recollect the case of an amiable young woman, who, although she had been before remarkable for the uniform cheerfulness and gaiety of her temper, became decidedly, and often deplorably dejected, in consequence of having for a length of time, been domesticated with an elderly friend who was of a desponding and melancholy cast. The contiguous atmosphere of an hypochondriacal, like that of a typhus patient, may, in a certain sense, be said to be impregnated with contagion."

The seventh Essay is "On excessive Study or Application of the Mind," which, though short, contains some excellent advice to literary gluttons. This Essay is followed by another equally brief, "On Vicissitude as a Cause and Characteristic Symptom of Intellectual Malady." Of the effect of transitions upon the mind, the following instance is given.

"I recollect the case of an unfortunate young man, who became a victim to the disastrous issue of a variety of mercantile adventures. The same blow which deranged his affairs, produced a disorder of his reason. His finances and his faculties fell together. The phantoms of imagination indeed survived, and seemed to hover over the ashes of his understanding. The demon of speculation, which had before misled his mind, now possessed it entirely. His projecting spirit, which was always more than moderately intrepid, took, in the maniacal exaltation of his fancy, a still bolder and sublimer flight. Some of his schemes reminded me of another madman that I had heard of, who planned, after draining the Mediterranean, to plant it with apple trees, and establish a cyder manufactory on the coast."

The ninth Essay is "On the Want of Sleep," as symptomatic and a cause of mental derangement; in which the author recommends the cold or the warm bath where dietetic opiates have failed.

The next Essay on "Intemperance," contains many valuable cautions with respect to the use and abuse of stimuli; well adapted to make a deep and salutary impression upon the minds of readers in general; but the following is not less deserving the attention of the faculty.

"Inebriety is not properly confined to the use of fermented liquors. The tipplers of laudanum are sots, although of another sort. There is something peculiarly plausible and seducing in this mode of fascinating the sensations. Opium does not in general, as wine is apt to do, raise a tumult of the feelings, or involve the intellect in clouds; but acts more like oil poured upon a tumultuous sea, which tends to allay the agitation of the billows, and induces an agreeable stillness and tranquillity. Instead of lowering man to a level with the beasts, it often invests him, for a time, with the consciousness and at least fancied attributes of a superior being; but he is soon stripped of his shadowy and evanescent prerogative, and is made to suffer all the horrors and humiliation of a fallen angel. The confessions of many a miserable hypochondriac, who has been in the habit of having recourse to opium for relief, justify this representation from the charge of caricature. Grievous as is the depression which takes place, as the second effect of fermented liquors, that which succeeds to the excitement produced by laudanum, is still more intolerable. It is of course a task less difficult to refrain from the former than the latter, when the latter has been for many years regularly applied to for temporary comfort or support, in a desertion or prostration of the spirits. The late Dr. Heberden was of opinion, that it is more easy to relinquish opium than wine, and therefore, in cases which may seem to require either the one or the other, he recommends the former in preferance to the latter. My own comparatively contracted experience would incline me, in the same circumstances, to give different advice.

"I have known only one case, in which an inveterate opium-taker has had resolution enough to dispel the charm which had long bound him to its use. This patient was in the custom of employing it in that concentrated form of the drug, which has received the appellation of the black drop. The dreadful sensations which he experienced for a considerable period, after having refrained from his wonted cordial, he was unable to express, any more than the gratitude which he felt towards his physician, for having strenuously and repeatedly, and at length successfully urged him to an abstinence from so delusive and bewitching a poison. When opium is employed as a remedy in cases of merely physical disease, it may not be liable to the same objection; although, even in that class of maladies, it ought to be in general reserved for occasions of urgency or peril. When used for a length of time without any considerable intervals, its bad effects upon the constitution will be found to accumulate, whilst its alleviating influence over troublesome and painful symptoms, becomes almost every day less observable."

From the danger of Intemperance, the author proceeds very appropriately to consider the injury occasioned by "the Excess of Abstinence;" for, as he observes, "we may be intemperately abstemious, as well as intemperately luxurious and indulgent. That degree of privation which is unnatural or unreasonable, proves no less destructive than superfluous and superabundant gratification."

This Essay is followed by one on "Morbid Affections of the Organs of Sense;" in which considerable attention is paid to the organ of vision; illustrated by some remarkable cases in the author's own practice. One of these we shall extract.

"During my attendance upon the Finsbury Dispensary, a remarkable instance of dimness of sight occurred, that had for some time previously been gradually approaching towards blindness, which, indeed, had actually taken place in one of the eyes. The patient first perceived the dimness the day after she had been frightened by witnessing a violent paroxysm of epilepsy, with which her husband had been attacked the preceding night. Since that time she had herself become, although not in the least so before, extremely liable to fits, and was apt to fall down insensible upon occasions of the slightest degree of agitation or surprise. Her dimness of sight seemed to consist, not in an injured state of the eye, but in a debility of the nervous system in general, that *appeared* more particularly in that delicate and exquisitely irritable part of it which is destined for the purposes of vision. The capacity of seeing with the eye that was not altogether blind, was intermittent, 'going and coming,' to use her own comparison, 'like the sun when a cloud passes over it.' The patient had likewise been subject to a deafness, that might be traced to the same circumstance as gave rise to her ophthalmic malady. Both symptoms had, in all probability, a common origin in nervous weakness or derangement."

In the thirteenth Essay, the opinion, or rather vulgar error, is successfully combated, "that madness, in any of its modifications, arises for the most part from an excess of intellectual vigour."

That "Physical malady may be the occasion of mental disorder," is proved in the next Essay by facts and arguments, while at the same time, the author very properly guards against any leaning towards the doctrine of materialism, which that position might be supposed to favour. We quote from this Essay, with great pleasure, the following reflections on the duty of moderation in scientific pursuits.

"Speculation with regard to the nature of the vital or intelligent principle in man, are involved in so much obscurity, as to allow greater scope for the display of a fertile imagination, than for the sober exercise of the reasoning faculty. The clouds in which this subject is enveloped, the rays of genius may illuminate, but cannot disperse. The unwarrantable boldness and decision with which many are apt to speak upon a question, which, from an incurable deficiency of data, admits of no satisfactory conclusion, argues a more than ordinary imbecility, rather than any superiority of understanding. Genuine intrepidity of every species, is naturally allied to modesty. There is a chaste and sober scepticism. When we profess that there is no moral evidence so immaculately clear, as to preclude all obscuration of doubt, we acknowledge merely the present imperfection and immaturity of our nature. A peremptory positiveness of opinion, as well as a rashness of action, is natural to the ardour and inexperience of youth; but diffidence gradually grows upon declining life. Unlimited dogmatism, in almost every case, affords suspicion of very limited information. In the degree in which our actual knowledge advances, we increase likewise our acquaintance with its comparative deficiency. As the circle of intellectual light expands, it widens proportionably the circumference of apparent darkness."

The Fifteenth Essay contains some good remarks on the polluted atmosphere of the metropolis, from which the author slides gradually into a brief notice of the very old, but not therefore true opinion, "that the gloomy month of November is peculiarly disposing to melancholy, and the favourite season of suicide." This proverbial reproach upon our climate, at that part of the year, is thus dismissed.

"The dark hues of the mind are not in general reflected from the sky; and the preternaturally exalted excitement of mania, soars in general above atmospheric influence. There are cases, indeed, in which the diseased apprehensions of an hypochondriac are relieved or aggravated by the changes of the weather; where, when the sun-shines, even his mind seems to be irradiated by its influence, and scarcely a cloud can obscure the face of nature, without at the same time casting a melancholy shade over his speculations."

The 16th Essay on "Dyspeptic and Hepatic Diseases," comprehends many valuable rules for regimen, and several hints equally worthy of professional observation and private attention. We were particularly pleased with the following remark, because it exactly conveys our own sentiments, and shews that the zeal of improvement may sometimes, even in the best concerns, be carried beyond the bounds of discretion, and that regulations and institutions, which we may think evil or unnecessary, may be both salutary and advisable.

"The observance of fasts is a wholesome form of superstition. The omission of them in the Protestant calender, was, perhaps, as it relates to health, an unfortunate result of the reformation. Though no longer regarded by us as religious institutions, it would be desirable that some of them at least should be still kept with a kind of sacred punctuality, as salutary intervals of abstinence, which give to the stomach a periodical holiday, and afford an occasional respite from the daily drudgery of digestion." 142.

This Essay is properly followed by one on "Palsy, Idiocy, Spasmodic and Convulsive Affections;" the perusal of which will yield both instruction and entertainment. Among the cases here neatly reported and yet accurately described, we are particularly struck with one which we shall give in the author's words:

"About two years ago, I met with a remarkable case, which strikingly exemplified the connexion and affinity that may exist between what are called 'bilious affections,' and those which belong more apparently and decidedly to the nervous system. The patient referred to, had, in consequence of a severe domestic privation, been seduced into habits of intemperance, which, for two years, seemed to have no effect but upon the liver, producing at nearly regular intervals of ten days, vomitings of bile, occasionally accompanied by a diarrhœa, which, when combined with the former, of course assimilated the disease to the character of cholera. For the considerable period above-mentioned, his only apparent complaint was what, in popular and fashionable language, is called the 'bile.' After the lapse, however, of somewhat more than two years from the commencement of his intemperate habits, without having received any precautionary or prefatory intimation, he was surprised by a seizure which paralyzed one half of his body, dividing it longitudinally into two equal sections, the one dead to all the purposes of sensation or voluntary motion, the other retaining the functions and privileges of vitality, although in some measure, of course, clogged and impeded by the impotent and diseased half to which it was united. When I saw him last, he had remained three years in this truly melancholy state. At least, during that time, he had experienced no important or permanent amelioration, nor any evident tendency towards the recovery of his corporeal powers. His mind also seemed to have shared in the paralysis. This was more particularly obvious in the lapses of his recollection. His memory had been maimed by the same blow which had disabled one side of his body. His recollection with regard to things, did not seem to be much impaired, but it was surprisingly so with regard to the denominations of persons or places. He has often forgotten the name of an intimate friend, at the very time that, with the most unaffected cordiality, he was shaking hands with him. Upon inquiry, it appeared that the pernicious habits of the patient were still persisted in; a circumstance which alone was sufficient to account for the uninterrupted continuance of his disorder.

"In this case, nothing could be more evident than that the bilious symptoms with which he was first affected, and the nervous complaints which succeeded, both originated from one source: and this may give a hint to those who are much troubled with the *bile*, as it is called, especially when it has been occasioned by the same means as in the instance just stated, that unless they seasonably reform their regimen, they may be at no great distance from a paralytic seizure." 158-162.

Dr. Reid speaks slightingly of the Bath waters as a remedy in paralytic cases, nor is he much more favourable to electricity; and, as he very properly observes,

"In the treatment of disease, it must appear desirable to effect the cure, when it is practicable, by means which act generally and impartially upon the body, rather than by those which operate, although not slowly, yet more immediately and with peculiar force, upon the delicate nerves and fibres of the stomach. The health, and of course comfort of man, depend in a principal degree, upon the due vigour of his powers of digestion, which by the inordinate or unnecessary use of drugs, has, in too many instances, been gradually impaired, and at length irrecoverably destroyed. This is apt to be the case, more especially with those fashionable hypochondriacs who are continually having recourse to the doses of pharmacy, in order to relieve the *ennui* of indolence, or to support the languor of an effeminate or enervated constitution. Such an existence as theirs may, out of courtesy, be called life, but it possesses none of life's privileges or its blessings." 176-177.

An ingenious but rather brief Essay on "Hereditary Madness," is the next in succession; and we should have been glad to have seen so excellent a disquisition upon a subject of great importance to society more extended; and our readers, no doubt, will be of the same opinion, after perusing the following remarks on the duty of celibacy in those who are radically of a morbid intellect.

"Nothing can be more obvious, than that one who is aware of a decided bias in his own person towards mental derangement, ought to shun the chance of extending and of perpetuating, without any assignable limit, the ravages of so dreadful a calamity. No rites, however holy, can, under such circumstances, consecrate the conjugal union. In a case like this, marriage itself is a transgression of morality. A man who is so situated, in incurring the risk of becoming a parent, involves himself in a crime, which may not improbably project its lengthened shadow, a shadow too which widens, in proportion as it advances, over the intellect, and the happiness of an indefinite succession of beings." 185-186.

The 19th Essay, on "Old Age," contains some good moral observations on the desire of longevity; but, disposed as we are to admire the penetration and judgment of the author, even in metaphysics, we cannot assent to his assertion, that "an old man is no longer susceptible of new ideas;" and that "his mind lives altogether upon the past." So far, indeed, as this may be said of the general character of climacterics with respect to the study of new arts and languages, we are of the same opinion: but many instances might be adduced of men who have been too much devoted to pleasure or business, to study in the prime of their years, but who have, in the decline of life, attained a competent degree of knowledge, and acquired a relish for inquiry which has been productive of the best consequences.

The Essay on "Lunatic Asylums" cannot fail to be read with avidity at a time like this, when these receptacles have, in an uncommon degree, excited the public attention and parliamentary investigation. This part of the work was written long before the subject had become a matter of general observation, and yet evils which have been developed by authority did not escape his examination, as appears from the following extract:—

"A heavy responsibility presses upon those who preside or officiate in the asylums of lunacy. Little is it known how much injustice is committed, and how much useless and wantonly inflicted misery is endured in those infirmaries for disordered, or rather cemeteries for diseased intellect. Instead of trampling upon, we ought to cherish, and by the most delicate and anxious care, strive to nurse into a clearer and brighter flame the still glimmering embers of a nearly extinguished mind.

"It is by no means the object of these remarks to depreciate the value of institutions which, under a judicious and merciful superintendance, might be made essentially conducive to the protection of lunatics themselves, as well as to that of others, who would else be continually exposed to their violence and caprice. But it is to be feared, that many have been condemned to a state of insulation from all rational and sympathising intercourse, before the necessity has occurred for so severe a lot. Diseased members have been amputated from the trunk of society, before they have become so incurable or unsound as absolutely to require separation. Many of the depôts for the captivity of intellectual invalids may be regarded only as nurseries for and manufactories of madness; magazines or reservoirs of lunacy, from which is issued, from time to time, a sufficient supply for perpetuating and extending this formidable disease—a disease which is not to be remedied by stripes or strait-waistcoats, by imprisonment or impoverishment, but by an unwearied tenderness, and by an unceasing and anxious superintendance.

"The grand council of the country ought to be aroused to a critical and inquisitorial scrutiny into the arcana of our medical prisons, into our *slaughter-houses* for the destruction and mutilation of the human mind."

The 21st Essay is "On the importance of counteracting the tendency to Mental Disease;" in which are these remarks upon one of the most important considerations arising out of the subject of insanity, as affecting the medical character in the reliance placed upon its testimony:

"Lucid intervals are a subject deserving of the very particular study of the legal, as well as the medical profession. There are, in fact, few cases of mania, or melancholy, where the light of reason does not now and then shine between the clouds. In fevers of the mind, as well as those of the body, there occur frequent intermissions. But the mere interruption of a disorder is not to be mistaken for its cure, or its ultimate conclusion. Little stress ought to be laid upon those occasional and uncertain disentanglements of intellect, in which the patient is for a time only extricated from the labyrinth of his morbid hallucinations. Madmen may shew, at starts, more sense than ordinary men. There is perhaps as much genius confined, as at large; and he who should court corruscations of talent, might be as likely to meet with them in a receptacle for lunatics, as in almost any other theatre of intellectual exhibition. But the flashes of wit betray too often the ruins of wisdom, and the mind which is conspicuous for the brilliancy, will frequently be found deficient in the steadiness of its lustre."

In the 22d Essay, the author treats of the use and abuse of "Bleeding;" which is admitted to be absolutely necessary in true pleurisy, but censured in strong terms, when indiscriminately resorted to in all cases of palsy and apoplexy.

On the subject of "Pharmacy," which is treated in the next Essay, the author very properly deprecates the practice of prolonging a medicinal course in cases of convalescence from acute disease; and the following observations, by the way of analogy, are equally deserving of the serious consideration of valetudinarians and practitioners.

"In the prescriptions of physicians, as well as in the preparations of cookery, a simplicity ought to be observed, which is in general, perhaps, not sufficiently attended to. A number of different dishes, which, separately taken, might be wholesome and nutritious, must altogether form a compound that cannot fail to have an unfavourable and disturbing effect upon the organs of digestion. In like manner, a glass of Port wine or a glass of Madeira, a draught of ale, or one of porter, might, in a state of debility or fatigue, for a time at least, invigorate and refresh; while if we take a draught, the same in quantity, but composed of all these different liquors, we shall find that, instead of enlivening and refreshing, it will nauseate and oppress. And yet something similar to this daily takes place in the formulæ of medical practitioners. A variety of drugs are often combined in the same recipe, each of which might be good, but the whole of which cannot. A mixture of corroborants or tonics, is not necessarily a tonic or corroborative mixture. A prescription ought seldom, perhaps, to contain more than one active and efficient ingredient; we should thus give that ingredient fair play, and by a competent repetition of trials might be able to ascertain, with tolerable correctness, its kind and degree of influence upon the constitution: whereas, out of a confused and heterogeneous mass, it is impossible for us to discriminate the individual operation of any one of the articles which compose it." 228-230.

In the 24th Essay, "Ablution" is considered in a similar manner with a decisive approbation of the use of cold water, as the means of preserving health, and of restoring it in particular diseases. But at the same time, the idea of superior advantages to be derived from sea-bathing is ridiculed with effect.

"Bodily exercise" is strongly recommended in the 25th Essay; in which, among many other acute remarks, we were particularly struck with the following:

"Improvements in the mechanism of modern carriages, by which they are made to convey a person from place to place, almost without giving him a sense of motion, may be one of the circumstances that have contributed to the increased prevalence of those maladies which originate in a great degree from a fashionable indulgence in lassitude and languor."

The next Essay has for its title, "Real Evils, a Remedy for those of the Imagination;" and, with the relation of some curious cases, it exhibits observations which may be considered as judicious hints for practice.

The last Essay in the volume is on the advantages arising from "Occupation;" the necessity of which is enforced by solid reasoning, apt illustration, and a singular case, with which we shall conclude our extracts, already sufficiently numerous.

"I was once consulted by a hypochondriacal patient, who had been the greatest part of his life a journeyman taylor, but who, by an unexpected accident, became unhappily rich, and consequently no longer dependent for his bread upon drudgery and confinement. He accordingly descended from his board; but Charles the Fifth, after having voluntarily descended from his throne, could net have regretted more severely the injudicious renunciation of his empire. This man, after having thrown himself out of employment, fell ill of the tedium of indolence. He discovered, that having nothing to do, was more uncongenial to his constitution, even than the constrained attitude, and the close and heated atmosphere in which he had been accustomed to carry on his daily operations. In one respect, however, the repentant mechanic was less unfortunate than the imperial penitent. It remained in the power of the former to reinstate himself in his former situation; which, after having resumed it, no motive could, a second time, induce him to reliquish." 260-262.

After so copious a view and minute an analysis of the present volume, any thing farther that we could say concerning it must be needless; the reader will see by the subjects treated, that the work is one of universal interest, because there is no human being, capable of thinking, who has not his seasons of mental depression or excessive irritation, who is not either called upon to watch over his own infirmities, or to commiserate those of others. In the extensive range of moral and actual ills, there is not one that is so frequently obtruded upon our feelings as nervous sensibility; and, therefore, a more benevolent office can hardly be undertaken, than that of pointing out the varieties of this Proteian malady, and the causes which tend to its ascendancy over the body and mind, till the grave closes upon the one, or reason is extinguished in the other.

